# DNA Damage Response Checkpoint Activation Drives KP1019 Dependent Pre-Anaphase Cell Cycle Delay in *S*. *cerevisiae*

**DOI:** 10.1371/journal.pone.0138085

**Published:** 2015-09-16

**Authors:** Lindsey A. Bierle, Kira L. Reich, Braden E. Taylor, Eliot B. Blatt, Sydney M. Middleton, Shawnecca D. Burke, Laura K. Stultz, Pamela K. Hanson, Janet F. Partridge, Mary E. Miller

**Affiliations:** 1 Department of Biology, Rhodes College, Memphis, Tennessee, United States of America; 2 Program in Biochemistry and Molecular Biology, Rhodes College, Memphis, Tennessee, United States of America; 3 Department of Biology, Birmingham-Southern College, Birmingham, Alabama, United States of America; 4 Department of Chemistry, Birmingham Southern College, Birmingham, Alabama, United States of America; 5 Department of Pathology, St. Jude Children’s Research Hospital, Memphis, Tennessee, United States of America; Florida State University, UNITED STATES

## Abstract

Careful regulation of the cell cycle is required for proper replication, cell division, and DNA repair. DNA damage–including that induced by many anticancer drugs–results in cell cycle delay or arrest, which can allow time for repair of DNA lesions. Although its molecular mechanism of action remains a matter of debate, the anticancer ruthenium complex KP1019 has been shown to bind DNA in biophysical assays and to damage DNA of colorectal and ovarian cancer cells *in vitro*. KP1019 has also been shown to induce mutations and induce cell cycle arrest in *Saccharomyces cerevisiae*, suggesting that budding yeast can serve as an appropriate model for characterizing the cellular response to the drug. Here we use a transcriptomic approach to verify that KP1019 induces the DNA damage response (DDR) and find that KP1019 dependent expression of *HUG1* requires the Dun1 checkpoint; both consistent with KP1019 DDR in budding yeast. We observe a robust KP1019 dependent delay in cell cycle progression as measured by increase in large budded cells, 2C DNA content, and accumulation of Pds1 which functions to inhibit anaphase. Importantly, we also find that deletion of *RAD9*, a gene required for the DDR, blocks drug-dependent changes in cell cycle progression, thereby establishing a causal link between the DDR and phenotypes induced by KP1019. Interestingly, yeast treated with KP1019 not only delay in G2/M, but also exhibit abnormal nuclear position, wherein the nucleus spans the bud neck. This morphology correlates with short, misaligned spindles and is dependent on the dynein heavy chain gene *DYN1*. We find that KP1019 creates an environment where cells respond to DNA damage through nuclear (transcriptional changes) and cytoplasmic (motor protein activity) events.

## Introduction

The eukaryotic cell division cycle is a highly regulated series of events supporting DNA replication, segregation and repair in response to a variety of intracellular and extracellular stresses. Agents capable of interacting with DNA can trigger the DNA damage response (DDR), which causes complex cellular signaling allowing for DNA repair and cell cycle delay [1, 2]. This delay can occur at several well-defined points in the cell division cycle, allowing time for DNA damage repair and a reduction in the potential to exacerbate DNA damage during DNA replication or chromosome segregation. In the absence of appropriate cell cycle delay and DNA repair, the accumulation of mutations or genomic rearrangements can impact many functions of the cell, up to and including cell viability, which can contribute to the development of cancer. So critical is the ability of cells to repair DNA and delay cell cycle progression that these responses remain highly conserved among eukaryotic species [3, 4].

DNA damaging agents, including the platinum-based drug cisplatin and carboplatin, are used in the treatment of solid tumors. Unfortunately, their clinical efficacy is hindered by dose-limiting toxicity to important organ systems, such as the gastrointestinal tract and peripheral nerves [5]. The inherent resistance of some primary tumors, as well as the acquired resistance of recurrent cancers, also limits the utility of this class of drugs [5]. Therefore, other metal-based chemotherapeutics have been developed with the aim of identifying drugs that reduce tumor size with minimal impact on healthy tissues. One promising candidate is the ruthenium complex *trans*-[tetrachlorobis(1*H*-indazole) ruthenate(III)], also known as KP1019. This drug has been shown to induce apoptosis in ovarian cancer and colon carcinoma cell lines *in vitro* [6, 7] and to shrink autochthonous tumors in rats [7–9]. KP1019 also maintains its efficacy against cell lines that are resistant to other chemotherapeutic agents [10]. Moreover, KP1019 has been shown to stabilize or reverse disease progression without dose-limiting toxicity in five of six evaluable patients in a Phase I clinical trial [6, 11]. Despite this progress, the signal transduction pathways that mediate the cellular response to KP1019 have not been adequately addressed.

Even though the molecular mechanisms by which KP1019 inhibits cell proliferation and induces apoptosis remain unclear, substantial evidence suggests that this drug damages DNA. For example, KP1019 has been shown to bind purine nucleotides [12] and DNA [13] in biochemical and biophysical assays. KP1019 treatment also increased tail-length in comet assays of colorectal carcinoma cells [7]. Furthermore, pharmacological inhibition of base excision repair and nucleotide excision repair increased the sensitivity of SW480 cells to the sodium-salt analog of KP1019 [6]. Studies in the budding yeast *Saccharomyces cerevisiae* also support KP1019’s genotoxicity. Specifically, KP1019 treatment increases rates of mutation and recombination in yeast, and genetic disruption of nucleotide excision repair, translesion synthesis, and recombination repair dramatically increase sensitivity to the drug [14]. KP1019 is capable of creating inter-strand crosslinks [13] resolution of which can produce double strand breaks. This idea is supported in *S*. *cerevisiae* given the profile of the DDR pathway sensitivity, which includes pathways known to be involved inter-strand crosslinks (ICL) resolution [14]. Given that cell cycle progression is exquisitely sensitive to DNA damage with the DDR-dependent delays occurring at multiple points in the cycle; it is interesting to note that KP1019 also induces a robust cell cycle delay in budding yeast, causing an accumulation of large budded cells [14] with an accumulation of 2C DNA content [15].

In the presence of DNA damage, checkpoint activation in *S*. *cerevisiae* depends on Rad9, a BRCT domain-containing protein [16–18], which promotes activation of effector kinases Chk1 (human Chk1 homolog) and Rad53 (human Chk2 homolog) [19–30]. Ultimately, activation of these pathways causes changes in gene expression to allow repair of DNA damage and appropriate cell cycle arrest. For example, the DDR response is marked by activation of *DUN1*, which encodes a serine/threonine kinase important for DNA damage-induced transcription [31, 32]. Dun1 functions in the same pathway as Rad9 and is required for DNA damaged induced de-repression of the Crt1 transcription factor, resulting in increased expression of a subset of genes, including the ribonucleotide reductase genes (*RNR1*, *RNR3*, *RNR4*) and *HUG1* [19, 20]. *S*. *cerevisiae’s* Rad9 dependent response to DNA damage, specifically double strand breaks, is thought to involve the Rad53 pathway and invokes a G2/M cell cycle delay via the Pds1-dependent stabilization of cohesin. In the presence of Pds1, cohesin maintains linkages between sister chromatids so that anaphase does not occur [33]. While the DDR in this case is clearly restricted to nuclear events [34], the complexities of this arrest point remain to be fully explained. For example, double strand breaks have also been shown to cause a DDR dependent triggering of cytoplasmic events that cause an increase in nuclear migration driven by spindle pole body movements in *S*. *cerevisiae* [35].

To more fully understand the cellular response to KP1019, we utilize the budding yeast *Saccharomyces cerevisiae* to characterize the KP1019-induced DDR. Genome-wide transcript analysis is consistent with the idea that KP1019 induces the DDR transcriptional response, and is confirmed by western blot analysis showing Dun1 dependent activation of target genes in response to KP1019. We find that the KP1019-induced DDR causes a robust pre-anaphase delay in cell cycle progression that is dependent on the Rad9 checkpoint. We also characterize an increase in Dyn1 dependent nuclear movement at the KP1019-induced arrest point, consistent with the idea that KP1019 induces double strand breaks in *S*. *cerevisiae*.

## Materials and Methods

YPD (dextrose) and synthetic complete (SC) media for yeast growth were prepared as described previously [14]. Yeast cells were transformed with DNA as described previously [36] or by using the Frozen-EZ Yeast Transformation II Kit (ZymoResearch T2001). Cis-Diammineplatinum(II) dichloride (cisplatin) was purchased from Sigma (P4394); KP1019 was provided by L. Stultz (Birmingham Southern College, Birmingham, AL). All KP1019 assays were done in the presence of synthetic complete media.

### Strains

The relevant genotypes and source of yeast strains used are shown in Table 1. Deletion strains were obtained from OpenBiosystems and are congenic with BY4741 for Mat a strains and BY4742 for Mat alpha strains. Commercially available deletion strains were used to obtain DNA template for PCR amplifications, and PCR deletion cassettes were used to construct deletions in marked strains for this study. When commercially available deletion strains were directly used for assays, they were first backcrossed at least two times to reduce the likelihood that phenotypes would be influenced by strain background variability. Cellular and nuclear morphologies were measured using *S*. *cerevisiae* strains where nuclear morphology can be tracked using mCherry marked histone Htb2 signal and spindle pole body position can be tracked using GFP marked Spc42 (kindly provided by M. Winey, U of Colorado). These strains were constructed through mating of Y984 (Mat a, *spc42ΔLEU2; trp-1-1*::*TRP1*::*Spc42GFP*(3x) *ade2-1*, *can1-100*, *his3Δ11*, *leu2-3*,*112*, *trp1-1*, *ura3-1* (kindly provided by Mark Winey, University of Colorado) and MMY313-2A (*Mat alpha HTB2-mCherry-spHis5 (HIS+)*, *ade-*, *trp- leu-*, *ura-*). MMY313-2A resulted from a cross between Y1522 (*Mat* *a*, *ade2-1*, *can1-100*, *his3D11*, *leu2-3*,*112*, *trp1-1*, *ura3-1*) and JB206-6C *(Mat alpha*, *ADE+*, *WHI5-GFP*::*kanMX HTB2-mCherry-SpHHIS5)* [37] (kindly provided by Jan Skotheim, Stanford University).

**Table 1:** Strains and relevant genotypes.

Strain	Genotype	Source
Y984	Mat a, *spc42ΔLEU2; trp-1-1*::*TRP1*::*SPC42GFP*(3x) *ade2-1 can1-100 his3Δ11 leu2-3*,*112 trp1-1 ura3-1*	M. Winey
Y1522	*Mat* *a*, *ade2-1 can1-100 his3D11 leu2-3*,*112 trp1-1 ura3-1*	M. Winey
YY4513	*ura3-1*::*URA3*::*TUB1-mCherry; spc42ΔLEU2; trp-1-1*::*TRP1*::*SPC42GFP(3x)*	M. Winey
JB206-6C	*Mat alpha WHI5-GFP*::*kanMX HTB2-mCherry-SpHIS5*	J. Skotheim
KT3279	*Mat a leu2 ura3 his3 PDS1-13Xmyc*::*kanMX*	K. Tatchell
MMY313-2A	*Mat alpha HTB2-mCherry-SpHIS5 ade- trp- leu- ura-*	This study
MMY318-5A	*HTB2-mCherry-SpHIS5 spc42*::*LEU2 trp-1-1*::*TRP1*::*SPC42GFP(3X)*	This study
MMY320	*HTB2-mCherry-SpHIS5 spc42*::*LEU2 trp-1-1*::*TRP1*::*SPC42GFP(3X) rad9Δ*	This study
MMY323	*HTB2-mCherry-SpHIS5 spc42*::*LEU2 trp-1-1*::*TRP1*::*SPC42GFP(3X) dyn1Δ*	This study
*RAD53*-TAP	S288C: (ATCC 201388: *MATa his3Δ1 leu2Δ0 met15Δ0 ura3Δ0*)	GE Dhamacon

### Microarrays

Yeast cultures grown in Synthetic complete media were grown to early log phase (OD600 between 0.2 and 0.3) and treated with either 40 or 80 μg/ml KP1019 for three hours. Cells were harvested by centrifugation, and cell pellets were stored at -80C in RNAlater (Qiagen RNeasy Kit, catalog number 74104). RNA was isolated using the RNAeasy Mini Kit (Qiagen RNeasy Kit, catalog number 74104) using the enzymatic lysis method. RNA was converted to cDNA and hybridizations were carried out using the 3DNA Array 900 labeling kit (Genisphere, catalog number W500180). Microarrays were obtained from the Genome Consortium for Active Teaching (http://www.bio.davidson.edu/GCAT/) and arrays were scanned through this consortium at Davidson College (Davidson, NC). Images were analyzed using MagicTool 2.1 (http://www.bio.davidson.edu/projects/MAGIC/magic.html) using total signal of adaptive circle with default parameters (min radius = 3 pixels; max radius = 8 pixels; threshold 50). Expression ratios were normalized and ratios found with a 3.5 or great fold difference between treated and untreated samples were analyzed further. Each array contained two genome copies and each experiment consisted of two arrays where samples fluorescent labels were switched to allow identification of preferential hybridization of capture reagent or cDNA based on capture sequences. Those samples whose % Difference ((T_1_-T_2_)/AVERAGE(T_1_,T_2_)*100 where T_1_ and T_2_ are the fold change values for the two trials) between dye reversal samples were greater than 50 or a relative fold change (range between T1 and T2/ the smaller value) greater than 3.5 were considered false. Preparation of all microarrays prior to scanning at Davidson College was carried out by students in an upper level genetics course taught by M.E.M. at Rhodes College. To avoid potential artifacts of array hybridization by students, genes were included in our list as induced or repressed if they met the above criteria in all replicates of the experiment. Significance of overlap between drug concentrations and previously published datasets was determined by exact hypergeometric probability tests with 6223 used as the total number of genes. The data discussed in this publication have been deposited in NCBI’s Gene Exprssion Onmibus [38] and are accessible through GEO Series accession number GSE 718005 (http://www.ncbi.nlm.nih.gov/geo/query/?acc=GSE71805).

### Alpha factor synchronization

Pds1 protein levels were measured using *S*. *cerevisiae* strain KT3279, which contains 12xmyc epitope tagged *PDS1* (kindly provided by K. Tatchell). Cultures were grown to log early phase (OD 600 0.2–0.3) and alpha factor was added to a final concentration of 0.6 mM until they were synchronized, as determined by morphology (generally a 2 hour exposure to alpha factor for this strain growing in synthetic complete media reaches over 80% unbudded/schmoo). Cells were harvested by centrifugation at 4°C and α-Factor was washed out with an equal volume of cold SC complete media, then briefly sonicated, washed in cold SC complete media, and then resuspended in prewarmed 30°C SC complete media with 80 μg/ml KP1019. Samples were collected every 20 min and assayed for Pds1 levels by immunoblotting. Synchrony was confirmed by determining the budding index.

### Protein extraction and immunoblotting

Total cellular protein lysates were obtained as described previously (Miller 2000). Immunoblotting was performed as described previously (Cross and Blake 1993). In the case of the Hug1 immunoblotting, 10–20% gradient tricine gels (Novex) were used to optimize visualization of the 5kDa Hug1 protein. In the case of Rad53-TAP detection, 4–20% Mini PROTEAN TGX precast gels (BioRad) were used. In all other cases, total cellular lysate were separated using 10–20% gradient Tris-Glycine Gels (Novex). The antibody used to detect Hug1 was kindly provided by M. Basrai (NIH) and previously described [39]. Pds1-myc epitope was detected using monocolonal α-myc E9010 (Santa Cruz Biotechnology). TAP tagged Rad53 protein was detected using rabbit polyclonal anti-TAP antibody (OpenBiosystems CAB1001). Loading controls consisted of either polyclonal anti-Nop1 antibody (EnCor Biotech),polyclonal anti-PGK antibody (Moelcular Probes), or nonspecific bands as indicated. Detection was done by enhanced chemiluminescence (ECL) with the super signal ECL Kit (Pierce) and visualized using an ImageQuant LAS4000 Luminescent Image Analyzer (GE Healthcare). For cell synchronization experiments, Pds1 band intensity was quantitated with ImageQuant software to determine maximum intensity of bands and values were adjusted to take into consideration differences in loading as determined by Nop1antibody. Time point 0 was set to a value of 1, and other bands normalized to this intensity. Detection of Rad53 protein was done as described previously [40] using now commercially available RAD53-TAP strain (GE Dhamacon). Early log phase cultures of Rad53-TAP yeast were incubated with indicated concentrations of KP1019 for one hour prior to protein isolation.

### IC50

Yeast strains MMY318-5A (wild-type) and MMY320 (*rad9* deletion) were grown to log early phase (OD 600 0.1), diluted 20 fold, and allowed to grow for 24 hours at 30C in the presence of a 2 fold serial dilution series of drug ranging from 160 μg/mL to 0.156 μg/mL of KP1019. Optical density readings at 600 nm were measured, graphed and used to carry out regression analysis using 4 parameter logistic on Sigma Plot. Each biological replicate was measured in triplicate, and the average IC50 reported in this paper reflects at least three independent biological replicates.

### Budding Index and Fluorescent Imaging Measurements

To determine budding index, cultures were grown to log early phase (OD 600 0.2–0.3). The culture was split, and the indicated amount of KP1019 was added. After three hours, the treated and untreated cultures were harvested by centrifugation and resuspended in 1X PBS. Formaldehyde was added to the cell suspension to a final concentration of 1.34% to fix the cells. To disrupt clumps fixed cells were sonicated for approximately 10 seconds at 4% intensity output (Sonicator Dismembrator Model 100, Fisher Scientific), then placed directly on a slide for budding morphology analysis. Cellular morphology was scored based on presence or absence of a bud. Buds were scored as large if they were larger than approximately two thirds the size of the mother cell, and small if they were smaller than approximately one third the size of the mother cell. Medium buds were sizes between these two categories. At least 300 cells were counted for each sample. Data for all least three independent replicates were collected.

Nuclear morphology and spindle pole body position in live cells were scored using HT2B-mCherry and Spc42-GFP, respectively. Images of live cells were captured using a LSM700 Confocal Imaging System (Zeiss). At least 300 cells were counted for each sample. Data for all independent replicates were collected. Distance between spindle pole bodies and longitudinal length of the cell was measured using ImageJ and normalized to account for cell size differences between treated and untreated samples. Spindle orientation was determined by calculating the angle of a line drawn between the two SPB to a line drawn along the long axis of the cell.

### Flow Cytometry

Histograms of DNA content were collected using a slightly modified method of Haase and Reed [41]. Cells were fixed in 70% ETOH and incubated in 10 mg/ml RNAse in 50 mM Tris pH 7.5 for 2 hours at 37C followed by incubation in a 5 mg/ml pepsin solution for 20 minutes at 37C. Cells were washed with 50 mM Tris pH7.5 and re-suspended in sytox green and sonicated prior to measurement of DNA content. Histograms were collected at the Flow Cytometry and Cell Sorting Shared Resource at St. Jude Children’s Research Hospital (Cancer Center Support Grant P30CA021765) on a BD Biosciences Fortessa analyzer, using 488nm excitation and detecting Sytox Green fluorescence at 510+/-10nm. Percentages of the population of cells in G0/G1, S, and G2/M as well as 2C/1C ratios were calculated using ModFit LT Version 4.1.7 (Verity Software House, Topsham ME).

## Results

### Transcriptional profile of KP1019 treated cells

When treated with the anticancer ruthenium complex KP1019, the budding yeast *S*. *cerevisiae* exhibits a robust dose-dependent cell cycle delay, resulting in accumulation of large budded cells that continue to increase in size, suggesting that they continue to actively metabolize [14]. To gain a better understanding of the cellular response to the drug at this arrest point we carried out a microarray based transcriptional analysis of cells exposed to either 40μg/ml or 80μg/ml KP1019 for three hours, conditions that result in reproducible accumulation of large budded cells [14]. Since three hours of treatment is sufficient to allow passage of untreated cells through the cell cycle, this exposure time resulted in synchronization of treated cells at the arrest point. We identified 50 genes that showed a 3.5 fold or greater drug dependent change in expression. Although there were differences between the sets of genes repressed at each drug concentration, the overlap was statistically significant for sets of induced (p = 2x10^-10^) and repressed (p = 4x10^-19^) genes (http://nemates.org/MA/progs/overlap_stats.html). This result suggests that the cells respond similarly to these two drug concentrations, which is consistent with the observation that both drug concentrations trigger cell cycle delay [14].

**Fig 1:**
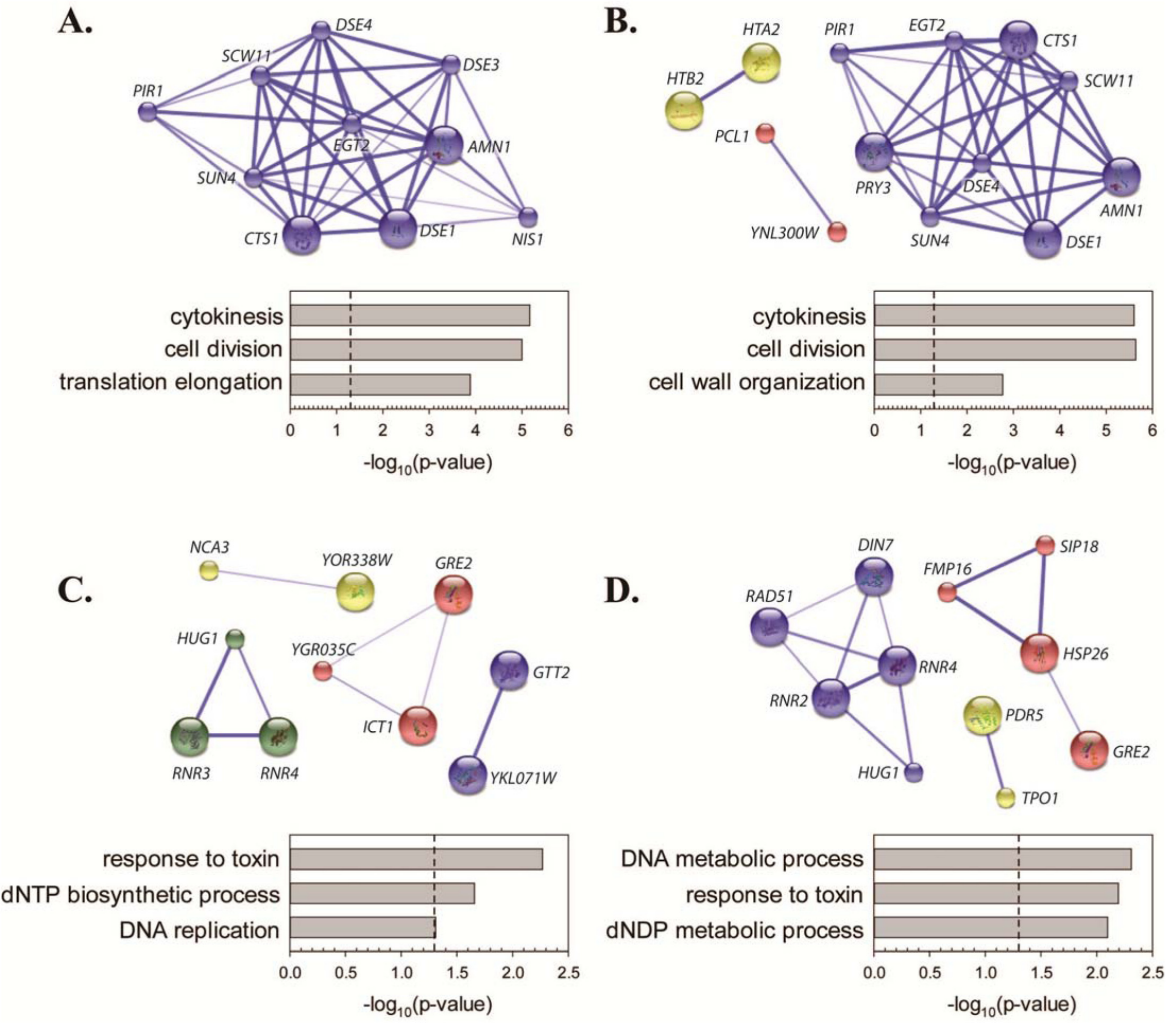
Transcriptomic analysis of KP1019-induced changes in gene expression. Early log phase wild-type yeast were treated with either 80ug/ml (A and C) or 40ug/ml (B and D) KP1019 for three hours. Extraction of RNA and microarray analyses were carried out as described in Materials and Methods. Genes repressed (A and B) and induced (C and D) by KP109 were assembled into clusters using the MCL algorithm on the Search Tool for the Retrieval of Interacting Genes/Proteins database (STRING; http://string-db.org/) [44]. Clusters were predicted with medium confidence and at an inflation level of 3 to allow for identification of a moderate number of clusters. Weak links and disconnected nodes are hidden for clarity. Node size indicates the presence (large nodes) or absence (small nodes) of structural data in the database. Within each cluster, the confidence of each inter-node connection is indicated by the width and intensity of the blue line. Graphs summarize biological process GO term analyses by DAVID. Redundant GO terms (like “dNTP metabolic process”) were omitted in the interest of clarity and brevity.

Analysis of repressed genes by Database for Annotation, Visualization and Integrated Discovery (DAVID; http://david.ncifcrf.gov/home.jsp) [42] revealed significant enrichment of genes involved in cytokinesis and cell division (Fig 1A and 1B). Genes in this functional category encode enzymes required to trigger cell separation after mitosis (for example, *CTS1* and *EGT2*), mother/daughter identify (*DSE* family proteins) and regulators of mitotic exit (*AMN1*, *NIS1*). Genes related to cell wall/cytokinesis were not induced by KP1019, though increased expression of genes important for phospholipid synthesis (*ICT1*) or cell wall synthesis during sporulation (*DIT4*) were observed. Given that KP1019-treated yeast accumulate as large budded yeast, it is possible that the expression of genes involved in the completion of cytokinesis would be low due to natural periodic expression through the cell division cycle. Consistent with this model, an exact hypergeometric probability test revealed significant overlap between genes repressed by KP1019 and the M/G1 group of genes defined by Spellman et al. [43] (p<0.0001 and representation factor >15 at both drug concentrations). Although cell cycle arrest is clearly a major contributor to drug-induced gene repression, only 75% of the genes repressed by 80ug/ml KP1019 were identified as cell cycle regulated by Spellman et al. [43], suggesting that the additional 25% of repressed genes may be down-regulated as more direct targets of drug action.

**Fig 2:**
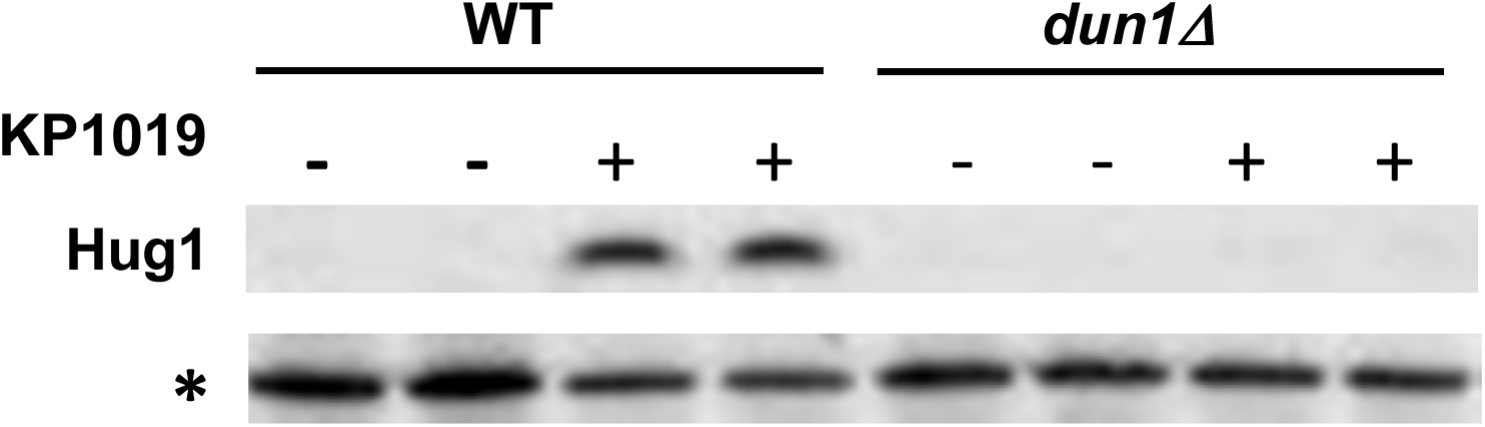
KP1019 induced *DUN1*-dependent expression of Hug1 protein and phosphorylation of Rad53. A. Early log phase wild-type (WT) and *dun1* deletion (*dun1Δ*) strains were treated with 80ug/ml KP1019 for three hours (+) or untreated (-) and assayed for Hug1 protein expression. Western blot analysis was carried out as described in Materials and Methods. Each sample was run in duplicate lanes and “*” indicates a non-specific band that serves as the loading control. B. WT cells were incubated with increasing concentrations of KP1019 for one hour and assayed for Rad53 protein phosphorylation. Western blots were carried out as described in Materials and Methods. The shift in position of Rad53 protein consistent with increased phosphorylation is indicated by Rad53-TAP-P, while unmodified Rad53 is indicated by Rad53-TAP.

Although the genes induced by KP1019 fell into multiple small clusters, as opposed to belonging primarily to one predominant cluster, analysis of induced genes by DAVID revealed significant enrichment of genes involved in response to toxin, nucleotide metabolism and DNA replication (Fig 1C and 1D). While these findings support previous reports of KP1019’s genotoxicity [7, 12, 14, 45] these data also suggest that KP1019 induces other stress response pathways. For example, induction of *GRE2* is consistent with Hog pathway activation [46], and elevated *HSP26* expression implicates the heat shock response [47, 48]. When considering average relative levels of expression as measured by microarray, the most induced gene in all replicates at both drug concentrations was *HUG1*. *HUG1* transcript is known to be regulated by DNA damage [31, 49], a result consistent with the induction of *RNR3* and *RNR4* in cells treated with 80 μg/ml KP0109 and *RNR4*, *RNR2*, *DIN7 and RAD51* in cells treated with 40 μg/ml KP1019 (Fig 1). *RNR1* did not meet the cutoff defining transcription induction at either drug concentration, and is thought to be regulated in response to DNA damage through a distinct mechanism from *RNR2-4* [50, 51]. Consistent with activation of the DNA damage response, transcriptomic analysis of induced genes also revealed signatures of cell cycle arrest. For example, an exact hypergeometric probability test revealed significant overlap between KP1019-induced genes and the group of M phase cell cycle genes identified by Spellman et al. [43] (p<0.02 and representation factor >5 for both drug concentrations). Moreover, there was significant overlap between Spellman et al.’s G2 phase genes and the genes induced by 80ug/ml KP1019. In addition, 16 genes that showed KP1019-dependent induction were not identified as cell cycle regulated by Spellman et al. Analysis of these non-periodic genes by DAVID showed significant enrichment of GO terms related to response to toxin and nucleotide synthesis, thus highlighting the primacy of the stress and DNA damage response pathways in controlling drug-induced transcriptional changes. We confirmed elements of the DDR transcriptional profile at the level of protein expression. Specifically, we assayed for a KP1019 responsive increase in Hug1 protein levels since *HUG1* transcript was consistently induced by KP1019. Hug1 has been previously established as a target of the DNA damage checkpoint normally regulated by Rad9 via activation of the Dun1 serine/threonine kinase [31, 32]. We confirmed KP1019 dependent Hug1 induction at the protein level, and found that induction of Hug1 by the drug was abolished by the deletion of the *DUN1* (Fig 2A). One mechanism by which the Dun1 pathway is activated involves the Rad53 protein kinase [52]. Phosphorylation of Rad53 is triggered by DNA damage, and results in a well-established mobility shift detectable by western blot analysis [53–55]. Consistent with these findings, we see a KP1019 dependent shift in protein of TAP tagged Rad53 protein (Fig 2B). These findings confirm that treatment of cells with KP1019 induces the Dun1 dependent pathway, as exemplified by increased expression of Hug1, supporting the idea that KP1019 induces the DDR. Additionally, other KP1019-induced genes can be linked to DNA replication stress or DDR (for example, *GTT2* [49], *PBY1* [49, 56], *FMP16* [49], and YKL071W [57]).

### KP1019 induces the *RAD9* DNA damage checkpoint

**Fig 3:**
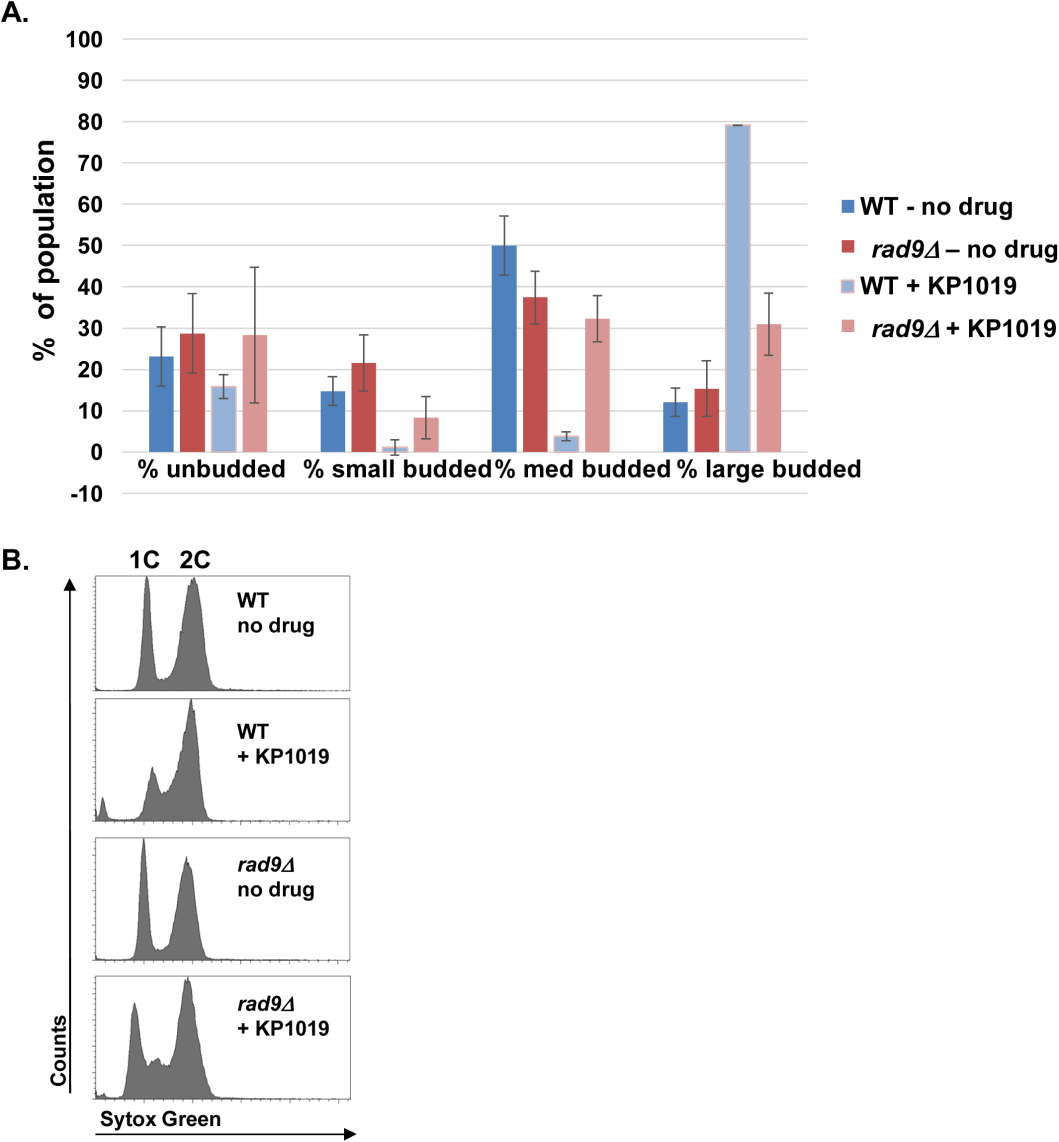
KP1019 induced cell cycle arrest requires *RAD9*. **A.** MMY318-5A (WT) and *rad9* deletion (*rad9Δ*) strains were assayed for budding index and DNA content. Early log phase cells were untreated (- KP1019) or treated for 3 hours with 80 mg/mL KP1019 (+ KP1019) and assayed for budding index or DNA content as described in Materials and Methods. **A.** Cells were fixed and scored for % of unbudded cells, small budded, medium budded and large budded cells within the population. Untreated samples are represented by blue (WT) and red (*rad9Δ*) bars, while treated samples are represented by light blue (WT) and light red (*rad9Δ*) bars. The average of three replicates is presented with 2XSE error bar. **B.** DNA content measured by flow cytometry using Sytox green based staining of nucleic acid as described in Materials and Methods. The x-axis represents increasing amounts of Sytox Green signal while the y-axis represents number of cells. Position of 1C and 2C DNA content peaks is indicated at the top of the Fig. panels. **C.** The average % cells in the population scored as G0/G1, S, and G2/M using ModFit analysis of samples exampled in (B). Untreated samples are represented by blue (WT) and red (*rad9Δ*) bars, while treated samples are represented by light blue (WT) and light red (*rad9Δ*) bars. The average of three replicates is presented with 2XSE error bars.

The transcriptional profile that results from exposing *S*. *cerevisiae* to KP1019 clearly supports the idea that KP1019’s genotoxic properties induce the DDR, but are the observed changes in gene expression and cell cycle progression causally linked to the DDR? Dun1 function in response to DNA damage has been shown to involve the Rad9 sensor protein [58]. In order to determine if KP1019-dependent cell cycle delay also requires Rad9 checkpoint activation, we determined the impact that deletion of *rad9* would have on cell cycle progression as measured by budding index, since bud emergence is concomitant with DNA replication (S phase) and the bud grows as cells progress through G2 and M phase. Deletion of *rad9* in the absence of drug does not significantly impact the distribution of cells showing large, medium, small or no buds (Fig 3A); consistent with the idea that in the absence of genotoxic damage the Rad9 checkpoint does not impact cell cycle progression. When comparing the wild type and *rad9* deletion strains that were exposed to KP1019, we found that approximately 30% of *rad9* deletion cells had large buds as compared to the near 80% observed in the WT *RAD9* strain (Fig 3A). Also, the percentage of small and medium budded cells fell in the treated WT strain, while the percentage of medium budded cells increased in the absence of *rad9*. These data are consistent a *RAD9* dependent accumulation of large budded cells in response to KP1019; supporting our hypothesis that KP1019 impacts cell division through the DDR signaling pathway. We note that the drug continues to induce a small increase in large budded cells, even in the absence of *rad9*, suggesting that the drug might continue to influence progression through the M phase transitions or cytokinesis in the absence of *rad9* (Fig 3A).

To confirm our findings, we assayed for cell cycle delay by measuring DNA content via flow cytometry. As expected, when comparing the treated and untreated WT samples, the height of the 1C peak drops relative to the height of the 2C peak when drug is present, suggesting a shift of the population to S (between 1C and 2C) or G2/M (2C) phases of the cell cycle. (Fig 3B). In the *rad9* deletion strain this shift is not as pronounced and the S phase population appears larger, suggesting that in the absence of *rad9*, the drug impacts cell cycle progression differently than in the WT strain. (Fig 3B). While the shift of the G0/G1 (1C) population is clear in these histograms, it is difficult to distinguish between S phase and G2/M phase cells. To attempt to clarify this aspect of the data, ModFit analyses were carried out to allow an estimation of the % of G0/G1, S, G2/M phase cells in the population (Fig 3C). At the point in the cell cycle where morphology and DNA replication are tightly coupled (exit from G1) our flow cytometry data and morphology data correlate very well–both show no impact of deleting *rad9* in the absence of genotoxic insult. Moreover when KP1019 is present we see a drop in G0/G1 cells that is in part dependent on *RAD9* (Fig 3). Interestingly, the *rad9* dependent shift revealed by the ModFit data occurs more so in the G2/M population and less so in the S phase population. These data are complicated by the fact that KP1019 appears to interfere with staining by the SYTOX Green nucleic acid probe so that the level of fluorescence of 2C vs. 1C cells is altered. Specifically, in the absence of drug, the WT and *rad9* deletion strains show a 2C/1C fluorescence ratio of approximately 1.90 +/- 0 and 1.91+/- 0.01 (Avg +/- 2X SE), respectively. These values are acceptably close to the expected 2C/1C fluorescence ratio of 2. In WT cells treated with KP1019 this ratio drops to 1.75+/-0.01 and in *rad9* deletion strains this ratio increases to 2.35+/-0.01. While reproducible, these shifts in the 2C/1C ratio were not expected, and suggest that the drug impacts aspects of DNA replication or segregation that influences whole DNA content per cell. These data might also suggest drug dependent variability in SYTOX Green staining or intercalation, so we hesitate to form strong conclusions based on the ModFit analysis alone.

Given that KP1019 induces the DDR dependent cell cycle delay in *S*. *cerevisiae*, we sought to determine if this response was important for drug tolerance by comparing drug sensitivity in wild-type and *rad9Δ* yeast. Deletion of *RAD9* increased sensitivity to the drug as measured by a decrease in IC50 from 34.4 μg/mL (1.7 μg/mL standard deviation) in wild-type to 11.1 μg/mL (0.35 μg/mL standard deviation) in the *rad9* deletion. Taken together, these data establish that KP1019 induces the well characterized *RAD9* dependent DNA damage response pathway in yeast resulting in a delay in the G2/M phase of the cell cycle, and that this response is important for survival after exposure to the drug.

### KP1019 induces mitotic arrest prior to the onset of anaphase

**Fig 4:**
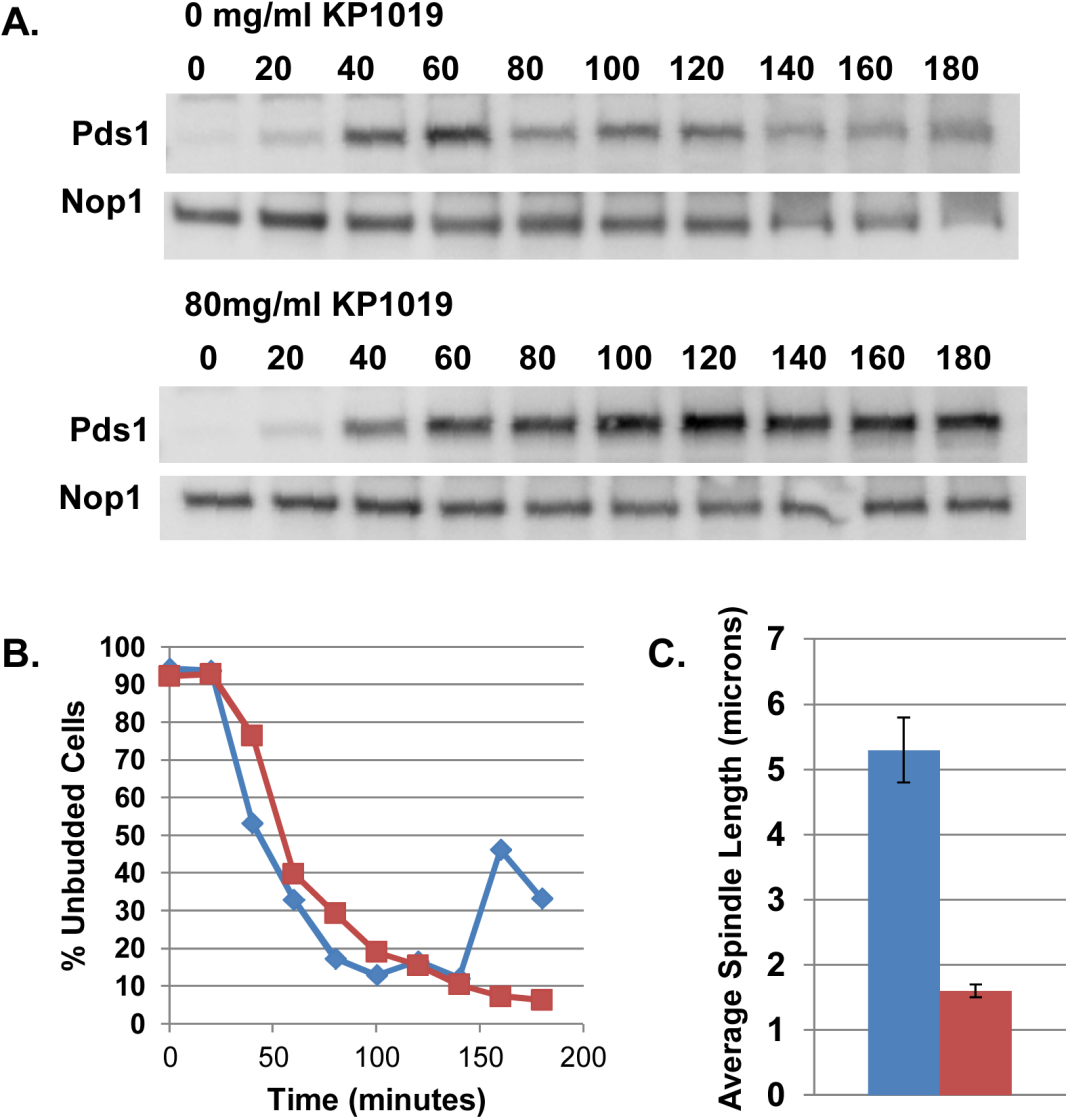
KP1019 induces a pre-anaphase cell cycle delay. **A and B.** Cells arrested in the G1 phase of the cell cycle by mating pheromone were released in the absence (0 mg/mL KP1019) and presence (80 mg/mL KP1019) of drug. Pds1-myc **(A)** and budding index **(B)** were measured every 20 minutes after release from G1. **A.** Pds1-myc epitope tagged protein expression levels assayed as cells progress through the cell division cycle. Antibody against the Nop1 protein is used as loading control. Normalized Pds1 band intensities are represented below each blot. **B.** Budding index (% of unbudded cells in the population) of cells used to measure Pds1 levels as cells progress through the cell division cycle. **C.** Distance between SPB in large budded cells that are untreated (blue bar) or treated with 80 mg/mL KP1019 for three hours (red bar) were determined as described in Materials and Methods. The average of three replicates is presented with 2XSE error bar.

We have observed that treatment of *S*. *cerevisiae* with KP1019 results in an increase of large budded cells (Fig 3A, [14]) and an increase of cells with 2C DNA content (Fig 3B, [15]), via a Rad9 dependent mechanism (Fig 3). To more finely characterize the point of KP1019 induced cell cycle delay, we determined the levels of Pds1 protein at the KP1019-induced arrest point. Degradation of Pds1 marks the onset of anaphase since it is required for the degradation of cohesin and subsequent chromosome segregation [59]. In order to measure the accumulation of Pds1 through the cell cycle, cells expressing myc-epitope tagged Pds1 were arrested in G1 using alpha factor and released as a synchronous culture in the presence of 80 μg/ml KP1019. Treated cells showed a sustained accumulation of Pds1 while untreated cells showed a reproducible reduction of Pds1 as they progress through the cell division cycle (Fig 4A and 4B). These data are consistent with the idea that KP1019 arrests cells with high levels of Pds1 and therefore prior to the onset of anaphase [26, 33].

To further support this finding, we characterized spindle pole body (SPB) position at the arrest point by using GFP tagged Spc42. Characterization of the SPB positions revealed that after exposure of a log phase culture to 80 μg/ml KP1019 for three hours, the majority of cells with large buds show duplicated SPBs in one cell body with an approximate average of 1.6 microns normalized distance between the two SPBs (Fig 4C). Because the KP1019 induced arrest results in cells of larger size [14] we normalized the distance between SPBs to the length of the longitudinal axis of each large budded cell to ensure that measurements of the distance between SPBs were comparable between treated and untreated cells. This normalization addresses concerns that distances between SPBs can be influenced by cell size, since size of the nucleus correlates with cell size [46], and yeast SPBs are embedded in the nuclear membrane in a closed mitosis. Strains expressing Spc42-GFP and Tub1-mCherry show microtubules spanning the distance between the two SPB (data not shown), suggesting that the spindle remains intact under these conditions. The observed distances between SPBs of KP1019 treated cells are consistent with established distances observed in pre-anaphase cells where chromosomes have attached to the spindle, created tension between the SPB, and not yet separated [60]. In contrast, we find that untreated large budded cells have on average a 5 micron distance between them, consistent with these cells having entered or completed anaphase (reviewed in [61]). Comparison of the angle between the axis of the two SPB positions and the longitudinal axis of each budding cell showed a wider distribution of angles, suggesting a more random pattern of SPB position than was observed for untreated cells (S1 Fig). Taken together, our Pds1 and SPB distance data is consistent with a pre-anaphase arrest point in response to KP1019.

### Exposure to KP1019 results in nuclear position defects

**Fig 5:**
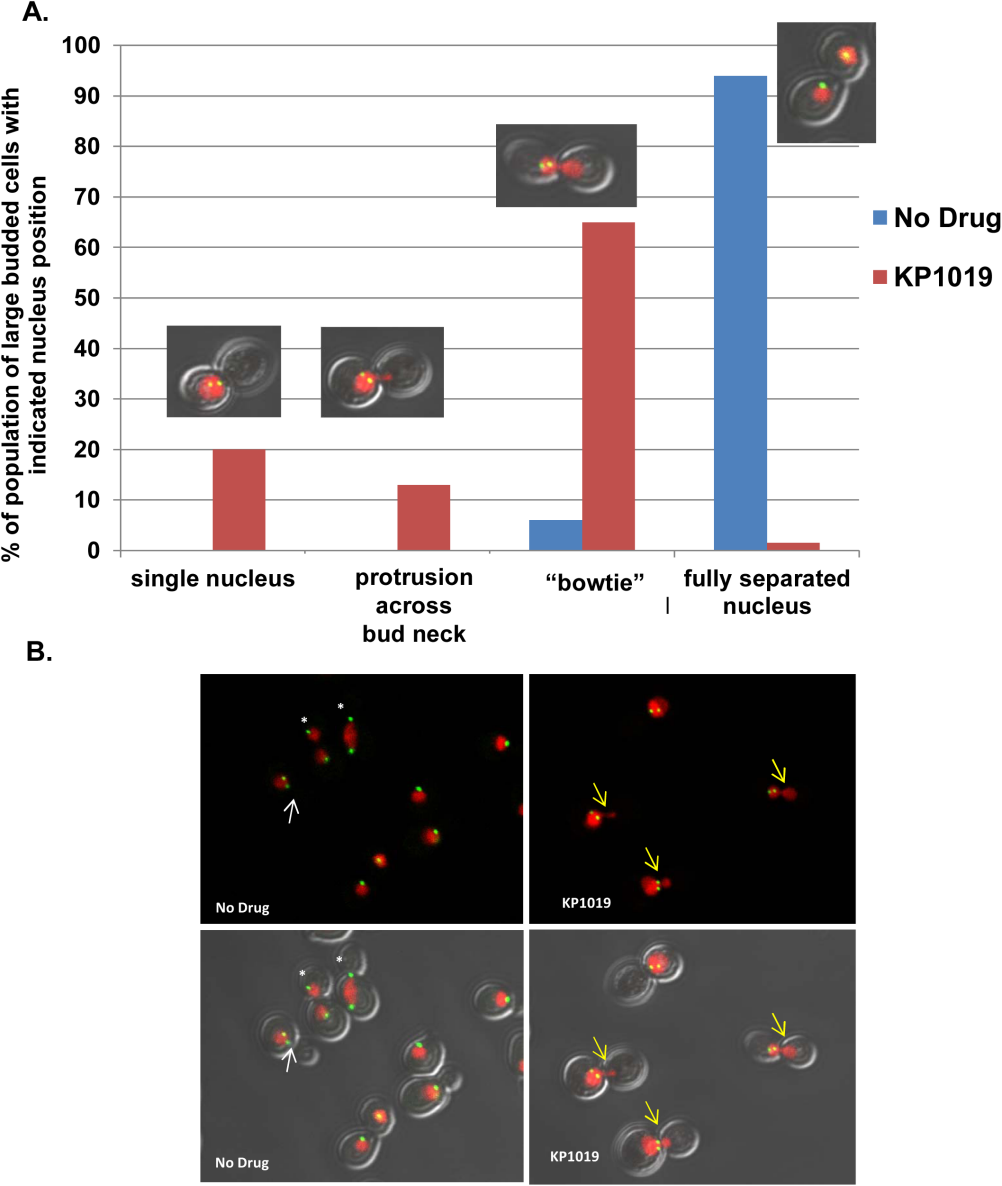
Nuclear Morphology in KP1019 treated cells. **A.** Early log phase wild-type yeast were treated with 80ug/ml KP1019 for three hours and nuclear morphology was determined as described in Materials and Methods. Large budded cells were scored on the position of the nucleus relative to the bud neck. Categories are indicated on the X-axis, and include a single nuclei positioned near the bud neck and not migrating across the bud neck (single nucleus), cells with some protrusion of nucleus across bud neck (protrusion across bud neck), nuclei spanning the bud neck (bowtie), and two separated nuclei positioned in each cell body (separated). Example image of treated cells from each category is provided. **B.** Representative images from the cytological analysis used to obtain data in Parts A showing varying levels of nuclear migration across the bud neck. Red signal is Histone H2-mCherry indicating nuclear position. Green signal is Spc42-GFP indicating SPB position. Arrows indicate cells with short spindles; the white color indicates an untreated cell with a single nuclei, and the yellow color indicates treated cells showing migration of nuclei into the daughter cell. White asterisk show fully separated spindle in untreated cells at varying points in anaphase. Note more random orientation of SPB bodies relative to the longitudinal cell axis exampled in treated cells (S1 Fig).

**Fig 6:**
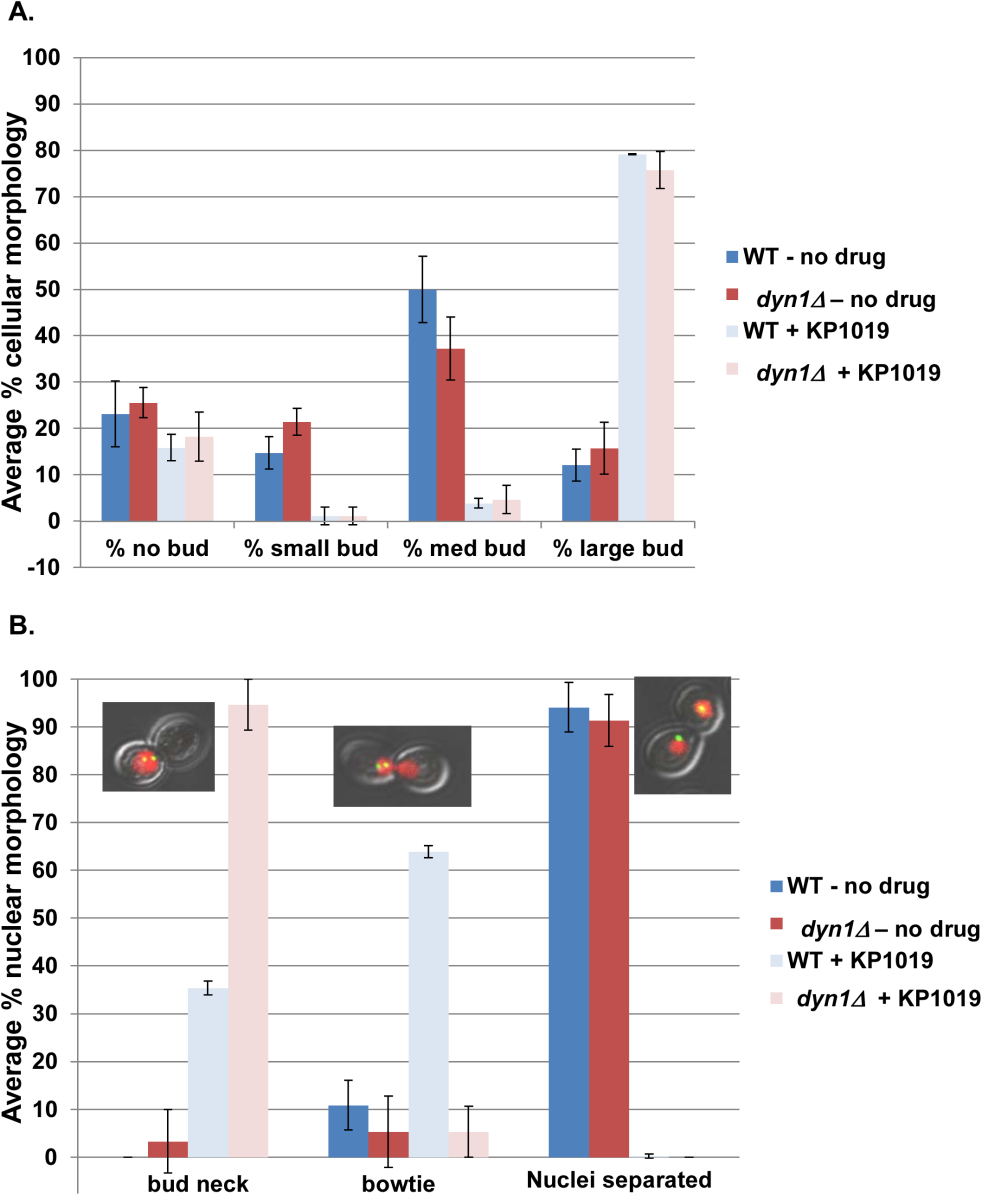
Dyn1 dependent KP1019 induced nuclear positions. Early log phase wild-type (MMY318-5A) and *dyn1* deletion (*dyn1Δ*) strains were treated with 80ug/ml KP1019 for three hours and assayed for budding index and nuclear position as described in Materials and Methods. **A.** *DYN1* deletion does not impact KP1019 induced cell cycle arrest. Untreated samples are represented by blue (WT) and red (*dyn1Δ*) bars, while treated samples are represented by light blue (WT) and light red (*dyn1Δ*) bars. The average of three replicates is presented with 2XSE error bar. **B.** KP1019 induced nuclear migration across the bud neck is Dyn1 dependent. Large budded cells were scored for the position of the nucleus relative to the bud neck. Categories are indicated on the X-axis, and include a single nuclei positioned near the bud neck, but not migrating across the bud neck (bud neck), cells with some protrusion of nucleus across bud neck (protrusion across bud neck), a bowtie phenotype (nuclei spanning bud neck), and two separated nuclei positioned in each cell body (Nuclei separated). Untreated samples are represented by blue (WT) and red (*dyn1Δ*) bars, while treated samples are represented by light blue (WT) and light red (*dyn1Δ*) bars. The average of three replicates is presented with 2XSE error bar.

Our previous work noted an interesting nuclear morphology in KP1019 arrested cells, where approximately 65% of large budded cells show an obvious nuclear migration across the bud neck and into the daughter cell ([14] and Fig 5). The mechanistic basis of the migration of the nuclei into daughter cells is curious; particularly given that our Pds1 and SPB distance data (Figs 3 and 4) are most consistent with a pre-anaphase arrest point. When considering the position of the SPB within these treated large budded cells, 82% show both SPBs in only one cell body, despite the observed nuclear migration across the bud neck (Fig 5). A similar phenotype coined “bowtie” nuclei has been observed in DDR mutants undergoing HO-dependent double strand breaks [35]. This work showed that the bowtie nuclear morphology correlates with increased dynamic SPB movement wherein both SPBs move between the mother cell and the bud. This SPB movement is dependent on the motor protein Dyn1 [35]. In order to test our hypothesis that the KP1019-induced bowtie phenotype requires the Dyn1 motor protein, we first needed to establish that KP1019 is able to induce cell cycle arrest in the absence of *DYN1*. Comparing WT and *dyn1* deletion strains we find similar cell cycle distribution shifts in response to drug, indicating that the ability of KP1019 to induce cell cycle arrest does not depend on *DYN1* (Fig 6A). To determine if *DYN1* is required for KP1019-dependent nuclear movement, we quantified the percentage of wild-type and *dyn1* deletion cells displaying the bowtie phenotype. We found that nuclear migration across the bud neck in large budded cells is dramatically curtailed from approximately 65% in wild-type to 5% in the absence of *DYN1* (Fig 6B). Therefore, the nuclear bowtie morphology that is observed after treatment with KP1019 is dependent on the motor protein Dyn1.

To determine if this phenotype is unique to KP1019, we assayed for bowtie nuclear morphology in response to the genotoxic platinum based chemotherapeutic cisplatin under conditions known to cause a G2/M large budded arrest with 2C DNA content [62]. Consistent with this previous work, we find that treatment with cisplatin causes cell cycle delay with an accumulation of large budded cells and 2C DNA content as measure by flow cytometry. Cisplatin is able to induce the bowtie nuclear morphology, though at a lower frequency than observed for KP1019 treated WT cells. After exposure to 1mM cisplatin for 3 hours, approximately 61% of the population of cells arrest with the large bud phenotype. Of these arrested cells, 34% show the bowtie phenotype. This modest accumulation of the bowtie phenotype in cisplatin treated cells was dependent on *DYN1*. 60% of the *dyn1Δ* cisplatin-treated cells arrested, but only 12% show the bowtie phenotype. Therefore, at a significantly lower frequency, the platinum based metal chemotherapeutic cisplatin also induces the Dyn1-dependent bowtie phenotype at the G2/M cell cycle delay.

## Discussion

When considering the anti-cancer agent KP1019, the paucity of well-defined and physiologically relevant signal transduction pathways that function in response to the drug hinders our ability to fully understand the mechanism of drug action. The identification of KP1019-dependent DDR signal transduction in yeast provides a novel finding that links KP1019 induced DNA damage to cell cycle arrest and drug sensitivity. Exposure of *S*. *cerevisiae* to KP1019 induces the Rad9 dependent DNA damage response (DDR) causing a change in the transcriptional profile of the cell and ultimately cell cycle arrest. This transcriptional response involves Rad53 phosphorylation and *DUN1* dependent expression of *HUG1*, demonstrating that the well-defined DDR is active in these cells (Figs 1 and 2). Microarray results suggesting activation of DDR have been further confirmed by reporter assays for the *RNR* genes (Hanson, personal communication). Deletion of the *RAD9* DDR checkpoint gene abrogates cell cycle arrest (Fig 3) as measured by both budding index and flow cytometry. We note that deletion of RAD9 does not return cell cycle profiles to normal suggesting that KP1019 also induces RAD9-independent arrest phenotypes. Deletion of *RAD9* increases sensitivity of yeast to KP1019, most likely due to an inability of the cells to repair DNA damage in the absence of the Rad9 checkpoint. Consistent with the activation of DDR by KP1019 in yeast, previous studies show increased sensitivity to mutations in DNA repair pathways including translesion synthesis, excision repair, and recombination based repair [14]. Taken together, these data fully support the fact that KP1019 induces DNA damage and that the DDR is critical for drug tolerance, supporting a physiologically relevant activation of the *RAD9* DDR checkpoint in response to KP1019.

Our finding that yeast treated with KP1019 arrest as large budded cells with elevated Pds1 levels and short spindles is consistent with a KP1019 induced pre-anaphase arrest (Fig 4), and Rad9 dependent DDR signaling. Interestingly, during the KP1019 induced cell cycle delay, we observed distortion and spreading of the nucleus across the bud neck, previously coined a bowtie phenotype. We find that in an actively dividing population of cells, less than 10% of large budded cells will show a bowtie phenotype, and in all cases these cells have SPB residing in both mother and daughter cells, suggesting that these cells are in fact progressing through anaphase. In contrast, approximately 75% of KP1019 treated large budded cells show migration across the bud neck and these cells contain both SPBs in one cell body (Fig 5). Moreover, the SPB orientation appears random when aligned with the longitudinal axis of the cell (S1 Fig). Although higher eukaryotes do not require nuclear movement through a constrained space (i.e. the bud neck), dynamic nuclear and centrosome movement is an integral part of appropriately regulated division. Specifically, the role of dynein dependent oscillations are highly conserved, particularly those aspects of dynein in nuclear oscillations and spindle dynamics [63–66].

Dyneins are AAA+ ATPases that support energy dependent motility toward the minus end of microtubules. Cytoplasmic dynein are implicated in the movement of cargo along microtubules, the positioning of the microtubule organizing center (MTOC in mammals and SPB in yeast), and organization of the microtubule networks associated with the cell cortex [67]. In budding yeast, the *DYN1* gene encodes the cytoplasmic dynein heavy chain [68, 69] and when deleted, yeast fail to move the spindle into the bud neck promptly, and cells enter anaphase with SPBs in one cell body. After sufficient cell cycle delay, the SPBs are able to eventually move across the bud neck in a dynein independent mechanism [68, 69]. Dynein dependent movement of the spindle and nucleus in budding yeast occurs during mitosis, but not during G1 indicating that this function is coordinated with the cell cycle. Cells arrested at the G2/M transition in response to DNA damage show accumulation of nuclei at the bud neck, but nuclei only rarely move across it. However, when checkpoint pathways (specifically Rad53 and Chk1) are mutated, a marked increase in the bowtie phenotype is observed in response to HO-induced double strand breaks and correlates with increases in Dyn1-dependent SPB movement [35]. While KP1019 dependent bowties occur in the context of wild-type checkpoint pathways (Fig 2), we speculate that this phenotype also results from KP1019 induced double strand breaks. We find that KP1019 induces an increase in Rad52-GFP foci and requires repair pathways consistent with the idea that KP1019 induces double strand breaks in yeast [14]. We find that KP1019 induced bowtie phenotypes are dependent on *DYN1*, suggesting that while KP1019 is able to trigger checkpoint activity required to impede chromosome segregation, it is unable to impede (and may possibly repress) those regulatory mechanisms that prevent nuclear migration [35, 70]. These data suggest that in addition to its genotoxic properties, KP1019 might be impacting the cytoplasmic response to DNA damage that influences spindle and nuclear movement(s). This impairment of spindle and nuclear dynamics may have clinical relevance, because poor prognosis in breast cancer patients is correlated with accumulation of tubulin modifications that reduce dynein/dynactic associated nuclear movement in yeast [71, 72]. These findings introduce the idea that cancer cells might be more sensitive to deregulation of dynein dependent nuclear movement that non-cancerous cells. We note that KP1019 induces enhanced nuclear movement, and speculate that if this aspect of KP1019 function is conserved it might account, in part, for the anti-tumor properties of the compound.

Given the possible novelty of the KP1019-dependent bowtie phenotype, we assayed for this phenotype in cells treated with the platinum based drug, cisplatin. Cisplatin is a widely used chemotherapeutic agent that shows greater patient toxicity than has been observed for KP1019 [6]. Understanding the mechanistic basis for this toxicity would provide exciting insight to chemotherapeutic drug design, our work with KP1019 shows similarities with studies addressing cisplatin function in yeast. For example, induction of the DDR as defined by *RNR* transcription has been established for cisplatin treated yeast [73] and DNA damage repair pathways have been implicated in drug tolerance [74]. Cisplatin is also able to induce an arrest at the G2/M transition prior to anaphase [62, 75]. However, when compared to KP1019 cisplatin is significantly less effective at inducing the bowtie phenotype in G2/M arrested cells. This suggests that while there are some similarities between the responses of budding yeast to these two genotoxic agents, significant differences can be observed. Moreover, our microarray analysis of yeast treated with KP1019 resulted in some overlaps and some differences in transcriptional profile reported. Specifically, cisplatin treated yeast also showed relatively few, 62, transcripts impacted including transcripts identified in our study as responsive to KP1019 (including *HUG1*, *SUN4*, *HO*, *SCW11* and *PIR1*) [76, 77]. Notable differences observed, including an absence of induction of DDR target genes *RNR2*,*3*,*4*; *HOG1* induced *GRE1*; and heat shock induced *HSP26*. Also, our microarray analysis failed to show KP1019 induced expression of the iron binding proteins Fit2 and Fit3 [78].

It is clear that transcripts induced in response to KP1019 include those associated with DDR/DNA metabolism. Induction of stress response-genes was also observed in these data, specifically of genes induced by exposure to toxins and replication stress. Among those genes implicated in stress responses, *GRE2* is induced by both drug concentrations tested. *GRE2* encodes a 3-methylbutanal reductase and NADPH-dependent methylglyoxal reductase [79, 80] whose transcription is regulated by a variety of stress responses [81] including the *HOG* [82] pathway [83]. We find an increase in steady state levels of Gre2 protein in response to treatment with KP1019 (data not shown). Singh et al recently reported a KP1019-induced *HOG* dependent stress response in yeast [46] which is consistent with our finding that at least one *HOG* pathway sensitive gene is induced by KP1019. We note that stress response genes with no known dependence on the *HOG* pathway were also identified in our analysis (for example, HSP26), demonstrating the novelty of our findings and the possibility that other stress response pathways are active in KP1019 treated cells. While not as prominent as previously reported [46], we observe an increase in small budded cells (indicating a delay in S phase) when treating asynchronous cultures and an increase in S phase cells as measured by flow cytometry of drug treated *rad9*-deletion cells (Fig 3C). These data are consistent with the idea that KP1019 has the potential to induce delay through S phase as well as at the G2/M phase transition [84]. ModFit analysis of the flow cytometry data showed a KP1019 dependent drop in the 2C/1C ratio suggesting that the drug impacts chromosome duplication and/or segregation. The 2C/1C ratio was influenced by Rad9, since deletion of *rad9* shifted the 2C/1C ratio to a higher value. A caveat to these findings is the possibility that the shift in 2C/1C fluorescence ratios reflects KP1019 interference with Sytox Green probe interactions with nucleic acid.

Genes repressed by KP1019 are primarily important for cell wall structure/function, specifically at the point of cytokinesis. Our observation that members of this functional category are down regulated with both drug concentrations is consistent with the idea that KP1019 induces a robust cell cycle delay as large budded cells. It would be sensible to expect a relative drop in expression in some genes involved in cytokinesis, since KP1019 arrests just prior to this event. For example, *AMN1* is expressed upon activation of the Mitotic Exit Network (MEN) [85, 86]. Based on the arrest point that we have characterized we would anticipate that the MEN has not been activated, so low *AMN1* expression relative to untreated log phase cells would be expected. However, many genes known to be expressed in response to cell cycle progression were not identified in our analysis, so we cannot rule out the possibility that KP1019, as opposed to cell cycle arrest, is triggering down regulation of these genes resulting in the observed defect in cell cycle progression, perhaps via a cytokinesis or spindle orientation checkpoint mechanism [87]. We note that while distinct from the arrest point described in our study, dynactin activity has been associated with checkpoints capable of monitoring cell wall synthesis in budding yeast that result in cell cycle arrest prior to SBP duplication [88].

Evidence is strong that a physiologically relevant target of KP1019 in eukaryotic cells includes DNA. Studies in *S*. *cerevisiae* further support the model that KP1019 triggers DNA damage. While specific types of DNA damage potentially induced by KP1019 remain unclear, our work is consistent with the idea that KP1019 triggers the formation of inter-strand crosslinks (ICL). Defects in DNA repair pathway genes, including loss of the ICL-specific *PSO2*, increase yeast sensitivity to KP1019. The ability of KP1019 to induce a Rad9-dependent pre-anaphase arrest with a lesser impact on S phase progression is consistent with the ability of the drug to induce double strand breaks [20, 35]. Our work also demonstrates a nuclear migration defect previously associated with repair of double strand breaks, the formation of which are part of the ICL repair pathway. These data do not rule out the possibility that DSBs are directly or indirectly (via nuclear or cytoplasmic targets) induced by other mechanisms, but the idea that KP1019 creates an environment where cells become sensitive to processes triggered by DSBs is intriguing. Future studies should more specifically address DSB induction by KP1019 and whether this occurs via ICL repair in yeast.

## Supporting Information

S1 Fig Orientation of spindle relative to longitudinal axis of cell. Box plot represents the upper quartile where 25% of the data is greater than this value and the lower quartile where 25% of the data is less than this value. Whiskers show largest and smallest angle values, not including outliers. Outliers are either more or less than 3/2 times the upper or lower quartile (respectively). Line internal to the box indicates the median angle. Treatment with drug results in a broader distribution of angles as compared to the untreated samples.
(TIF)
