# Correction: DNA Damage Response Checkpoint Activation Drives KP1019 Dependent Pre-Anaphase Cell Cycle Delay in *S*. *cerevisiae*

**DOI:** 10.1371/journal.pone.0141518

**Published:** 2015-10-28

**Authors:** PLOS ONE Staff

Fig 2 is incorrect. Please see the correct Fig 2 here. The publisher apologizes for the error.

**Fig 2:**
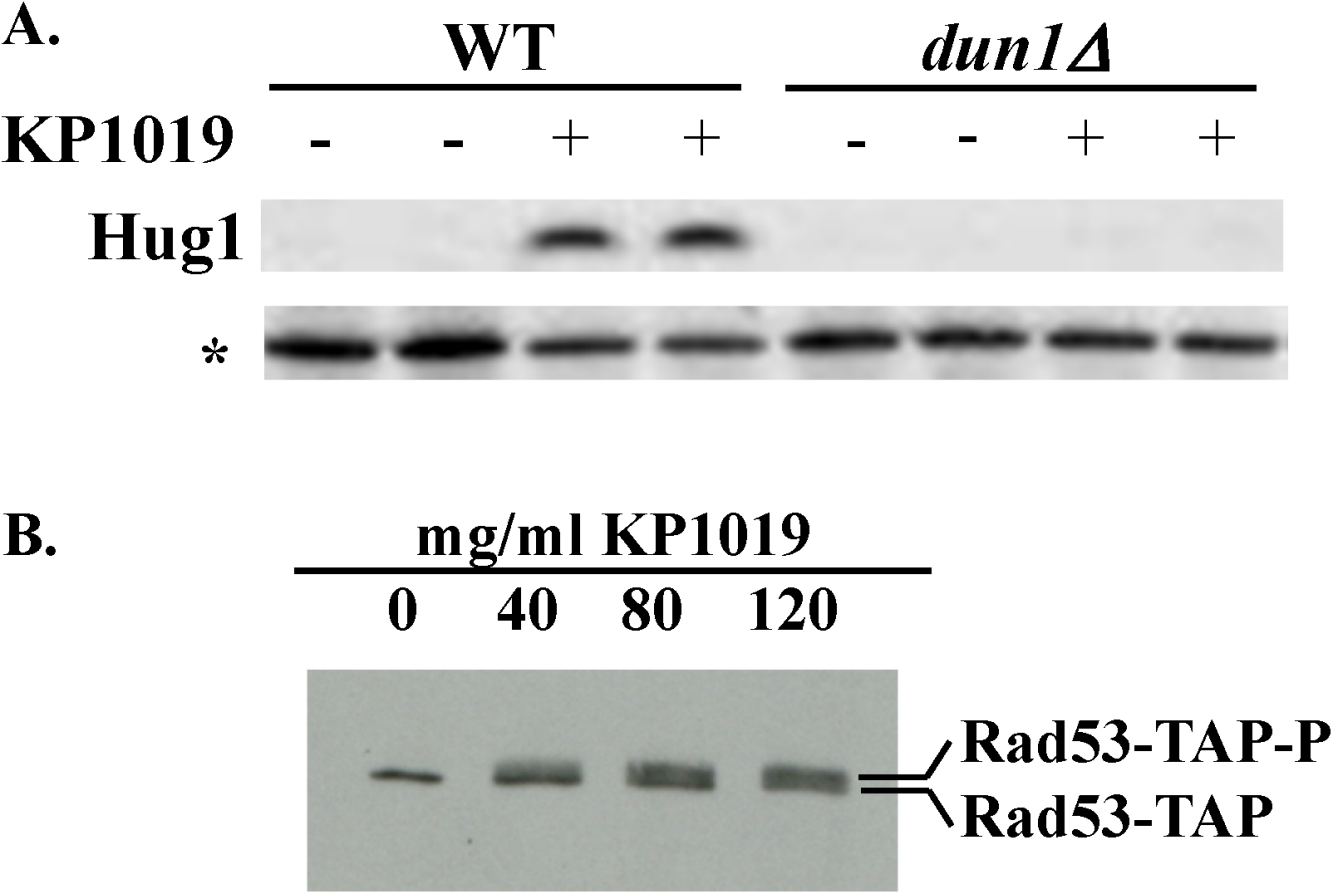
KP1019 induced DUN1-dependent expression of Hug1 protein and phosphorylation of Rad53. A. Early log phase wild-type (WT) and dun1 deletion (dun1Δ) strains were treated with 80ug/ml KP1019 for three hours (+) or untreated (-) and assayed for Hug1 protein expression. Western blot analysis was carried out as described in Materials and Methods. Each sample was run in duplicate lanes and “*” indicates a non-specific band that serves as the loading control. B. WT cells were incubated with increasing concentrations of KP1019 for one hour and assayed for Rad53 protein phosphorylation. Western blots were carried out as described in Materials and Methods. The shift in position of Rad53 protein consistent with increased phosphorylation is indicated by Rad53-TAP-P, while unmodified Rad53 is indicated by Rad53-TAP.
