# Supplementary figures and images for: DNA Damage Response Checkpoint Activation Drives KP1019 Dependent Pre-Anaphase Cell Cycle Delay in S. cerevisiae
Source: PLoS One. 2015 Sep 16;10(9):e0138085. doi: 10.1371/journal.pone.0138085 (PMC4572706; doi:10.1371/journal.pone.0138085)

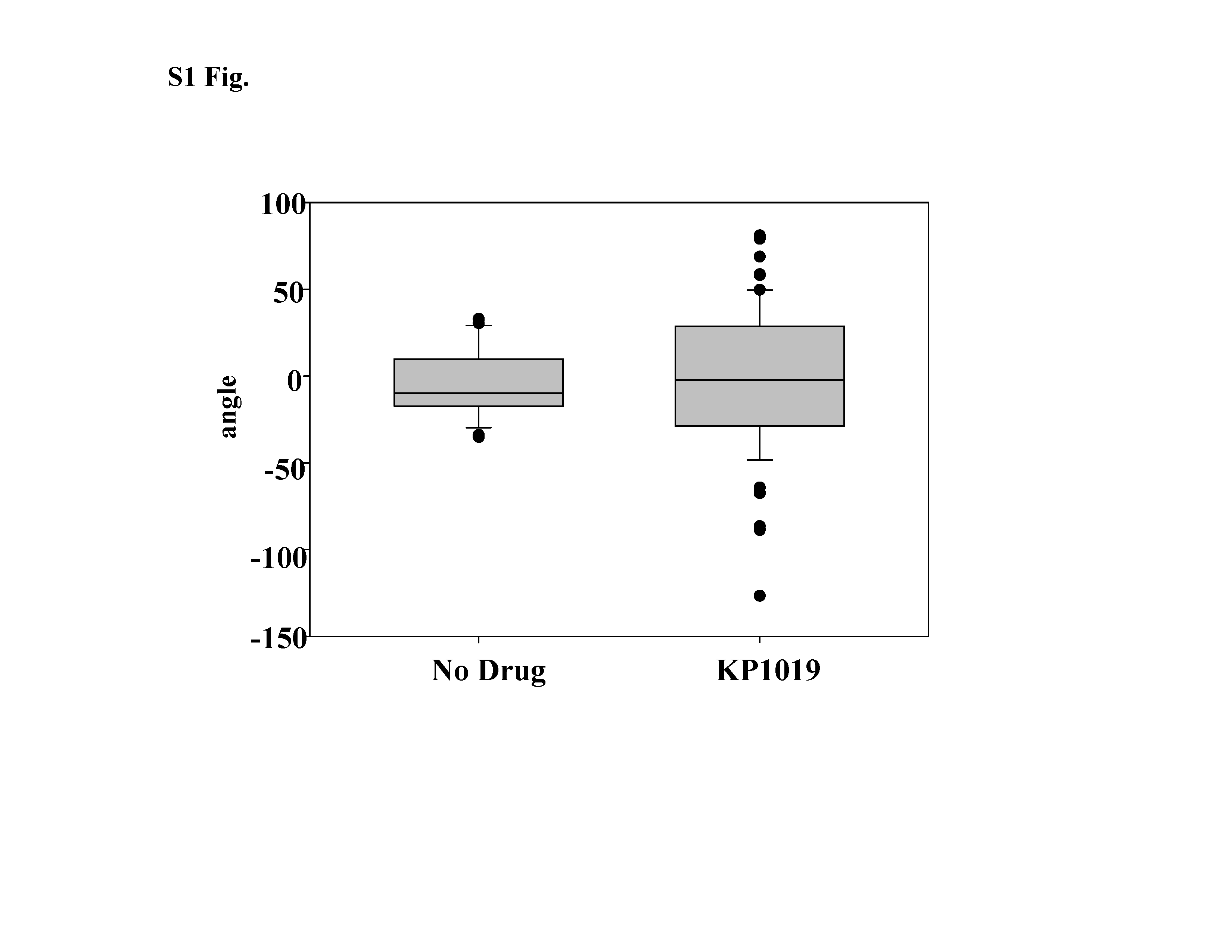

Supplement: S1 Fig — Box plot represents the upper quartile where 25% of the data is greater than this value and the lower quartile where 25% of the data is less than this value. Whiskers show largest and smallest angle values, not including outliers. Outliers are either more or less than 3/2 times the upper or lower quartile (respectively). Line internal to the box indicates the median angle. Treatment with drug results in a broader distribution of angles as compared to the untreated samples. (TIF) [file pone.0138085.s001.tif]
